# Bilateral Symmetrical Brain MRI Findings in Acute Necrotising Encephalopathy Type 1

**DOI:** 10.3390/children12080974

**Published:** 2025-07-24

**Authors:** Alexander T. Hoppe, Twinkle Ghia, Richard Warne, Peter Shipman, Rahul Lakshmanan

**Affiliations:** 1 Medical Imaging Department, Perth Children’s Hospital, Nedlands, Perth 6009, Australia; alexander.hoppe@health.wa.gov.au (A.T.H.); richard.warne@health.wa.gov.au (R.W.); peter.shipman@health.wa.gov.au (P.S.); 2 Neurology Department, Perth Children’s Hospital, Nedlands, Perth 6009, Australia; twinkle.ghia@health.wa.gov.au; 3 Perth Radiological Clinic, Subiaco, Perth 6008, Australia; 4 Centre for Neuromuscular and Neurological Disorders, Perron Institute, UWA Medical School, Perth 6009, Australia

**Keywords:** acute necrotising encephalopathy, acute necrotising encephalopathy type 1, RAN binding protein 2, MRI, lateral geniculate bodies, claustrum, subthalamic nuclei, thalami, mamillary bodies

## Abstract

**Background:** Acute necrotising encephalopathy (ANE) is a rare and severe type of encephalopathy with bilateral symmetrical brain lesions, often following a viral prodrome. ANE type 1 (ANE1) is a disease subtype with a predisposing mutation in the gene encoding RAN binding protein 2 (*RANBP2*). **Methods:** We report a case of a 3-year-old girl with clinical symptoms of ANE and brain MRI findings suggesting ANE1, which was subsequently confirmed by genetic analysis. **Results:** MRI of the brain demonstrated symmetrical high T2/FLAIR signal changes in the lateral geniculate bodies, claustrum, ventromedial thalami, subthalamic nuclei, mamillary bodies, and brainstem, with partly corresponding diffusion restriction, as well as additional haemorrhagic changes in the lateral geniculate bodies on susceptibility weighted imaging. Genetic analysis revealed a heterozygous pathogenic variant of the *RANBP2* gene. With immunosuppressive and supportive treatment, the patient fully recovered and was discharged after 10 days in the hospital with no residual symptoms. **Conclusions:** Recognition of the characteristic MRI findings in ANE1 can facilitate a timely diagnosis and enhance the clinical management of the patient and their relatives, especially given the high risk of disease recurrence.

## 1. Introduction

Acute necrotising encephalopathy (ANE) is a rare type of encephalopathy characterised by fulminant neurological symptoms and multifocal symmetrical brain lesions, often preceded by a viral prodrome [1]. It was first described by Mizuguchi et al. in 1995 [2]. Pathologically, the lesions show oedema, haemorrhage, and necrosis, with a characteristic absence of inflammatory cells in the affected brain parenchyma [3].

While most cases are sporadic, familial cases of ANE have been described and found to be associated with a causative gene mutation in RAN binding protein 2 (*RANBP2*), suggesting a genetic predisposition to the disease and its associated immune dysregulation [4,5]. *RANBP2* is a nuclear pore protein expressed in all tissues, with a wide range of intracellular functions [6]. ANE associated with a *RANBP2* mutation is subtyped as ANE1, also referred to as infection-induced acute encephalopathy 3 (IIAE3) [4,5]. It remains unclear how exactly *RANBP2* gene mutations contribute to disease development. They may directly impact the viral infection process or act downstream in the activation and modulation of immune cells and the production of cytokines in a “cytokine storm” [4,5].

The diagnostic criteria of ANE1, as proposed by Neilson et al., suggest that any of the following criteria must be met in addition to the diagnostic criteria for ANE: (I) a familial history of neurological symptoms which might be para-infectious, (II) recurrent encephalopathy following fever, and (III) additional magnetic resonance imaging (MRI) changes in one of the following: medial temporal lobes, insular cortices, claustra, external capsules, amygdalae, hippocampi, mammillary bodies, and the spinal cord [1,2,7,8]. More recently, the lateral geniculate bodies specifically, which are nuclei and an anatomical part of the thalamus, have been reported to be commonly involved in this subtype [9].

The prognosis of ANE varies from complete recovery to death, but morbidity and mortality are substantial, with mortality rates of up to 30% and neurological sequelae frequently seen in survivors [10,11,12]. In a study that included *RANBP2*-mutation-associated cases of ANE, a positive *RANBP2* status was predictive of relapse but not predictive of the overall outcome [13]. Recurrence is a hallmark feature of ANE1, and about 50% of patients will have at least one further episode [7].

## 2. Case Report

A 3-year-old female presented to a regional hospital with a 3-day history of cough, vomiting, fever, and lethargy; she was not walking and not talking. She had initially presented 24 h prior with a fever and cough and was treated for croup with a stat dose of dexamethasone and discharged home. A nasopharyngeal swab was positive for Influenza A. Her C-reactive protein level was normal, as were her liver enzymes, renal function, and electrolytes. The patient was started empirically on acyclovir, ceftriaxone, vancomycin, oseltamivir, and dexamethasone (4 mg orally every 12 h), before being urgently transferred to our hospital.

The patient’s past medical history was significant only of recurrent urinary tract infections. The perinatal and developmental history was unremarkable. There was a positive family history of encephalitis: the maternal grandmother had suffered an episode in the first year of life from which she fully recovered, and a maternal cousin had died from encephalitis.

On arrival, her examination revealed fluctuating alertness with mild encephalopathy and low tone with normal reflexes and truncal and peripheral ataxia. There was no meningism. Her vital signs were normal. She was managed symptomatically on the ward while awaiting further investigations. Further work up revealed normal creatine kinase, ammonia, urine organic, and amino acid screens as well as plasma amino acids. Her cerebrospinal fluid (CSF) was acellular. Her CSF protein level was raised (0.73 g/L, normal 0.1–0.35 g/L). Her CSF glucose level was normal, as were her CSF amino acids, lactate and pyruvate levels. Oligoclonal bands were negative. CSF neopterin levels were raised. CSF multiplex polymerase chain reaction tests for viral and bacterial pathogens were negative. The erythrocyte sedimentation rate was mildly raised (21 mm/h, normal 3–13 mm/h). NMDA, aquaporin-4, and MOG antibodies as well as ANA were negative. Her electroencephalogram showed background slowing in keeping with mild encephalopathy.

**Figure 1:**
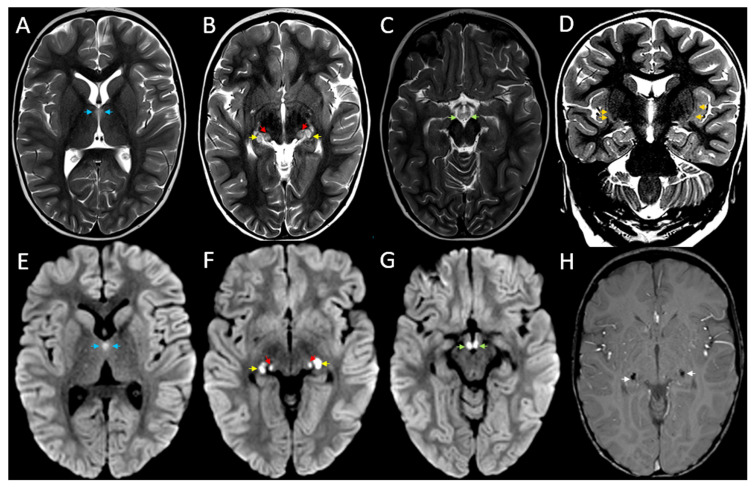
Bilateral symmetrical MRI findings in acute necrotising encephalopathy type 1. The 3T MRI images including axial T2 weighted imaging (**A**–**C**), coronal T2 weighted imaging (**D**), axial diffusion weighted imaging (**E**–**G**), and axial susceptibility weighted imaging (SWI) (**H**). There is T2 hyperintensity and corresponding diffusion restriction demonstrated within the bilateral ventromedial thalami ((**A**,**E**), blue arrows), lateral geniculate bodies ((**B**,**F**), yellow arrows), subthalamic nuclei ((**B**,**F**), red arrows), and mamillary bodies ((**C**,**G**), green arrows). T2 hyperintensity is also demonstrated within the bilateral claustrum ((**D**), orange arrows). The lateral geniculate bodies show evidence of haemorrhage on SWI ((**H**), white arrows).

The MRI of the brain performed on day 2 of hospital admission demonstrated symmetrical high T2/FLAIR signal changes with corresponding diffusion restriction in the ventromedial thalami, lateral geniculate bodies, subthalamic nuclei, and mamillary bodies. The lateral geniculate bodies showed associated susceptibility artefact on susceptibility weighted imaging (SWI), in keeping with the haemorrhagic change. Symmetrical high T2/FLAIR signal changes were also observed bilaterally in the claustrum. More subtle and diffuse high T2/FLAIR signal changes were further seen in the brainstem, primarily the midbrain. Overall, the MRI findings, summarised in Figure 1, were suggestive of an acute necrotising encephalopathy as can be seen in the setting of an Influenza A infection, with the symmetrical involvement of the claustra and the lateral geniculate bodies especially raising the possibility of an underlying *RANBP2* gene mutation. Subsequent genetic analysis revealed a pathogenic heterozygous variant in *RANBP2* (c.1754C>T: p.Thr585met), thereby confirming the diagnosis of ANE1.

The ANE severity score (ANE-SS) was three (moderate) due to the presence of elevated CSF protein and brainstem involvement on imaging [14]. The patient was treated for 5 days with intravenous methylprednisolone (20 mg/kg daily), started shortly after the MRI on day 2 of hospital admission. She improved gradually over the next days and was discharged home after 10 days with no residual symptoms. A repeat MRI around 3 months later in ongoing full clinical remission showed complete resolution of the previously demonstrated T2/FLAIR signal changes and diffusion restriction, with residual blood products within the lateral geniculate bodies demonstrated on SWI.

## 3. Discussion

ANE is a rare disease with substantial mortality and morbidity, and timely diagnosis and adequate management are crucial. While most cases are sporadic, knowledge of the likely underdiagnosed recurrent and/or familial disease form ANE1, related to a mutation in the *RANBP2* gene, is essential.

The pathogenic heterozygous variant in the *RANBP2* gene (c.1754C>T: p.Thr585met) found in our patient is the most commonly reported variant associated with recurrent or familial acute necrotising encephalopathy [5,15]. Its presence has also been described in unaffected individuals, suggesting incomplete penetrance, which is estimated to be around 40% [7,16].

There are proposed diagnostic criteria for ANE and ANE1, and since clinical symptoms of ANE1 significantly overlap with those of isolated ANE, the patient and familial histories as well as MRI findings assume particularly important roles in identifying cases of this disease subtype [1,2,7,8]. The Neilson criteria include possible MRI findings in ANE1, namely the additional involvement of the medial temporal lobes, insular cortices, claustra, external capsules, amygdalae, hippocampi, mammillary bodies, and spinal cord [7,8]. More recently, lateral geniculate bodies specifically have been reported to be commonly involved in this condition, as was also the case in our patient [9]. A study including cases of *RANBP2*-mutation- and/or influenza-associated acute necrotising encephalopathy, however, did not reveal significant differences in MRI findings for positive versus negative *RANBP2* status, with the small study cohort of 20 patients limiting the observation’s validity [13].

Wong et al. described a significant correlation between a proposed MRI scoring system, ranging from 0 to 4, and the clinical outcome in 12 patients with ANE, based on lesion location and the presence of haemorrhage and cavitation, assessed on both an initial and a follow-up MRI at a mean of 62 days after disease onset [3]. Our follow-up MRI took place 101 days after disease onset, and the MRI score according to Wong et al. in our case was two due to brainstem involvement and the presence of haemorrhagic change, which is on the lower side and thus in line with the authors’ observation of better clinical outcomes in patients with lower MRI scores [3]. Applying the same scoring system in 41 patients with ANE, Zhu et al. also found a significant difference between patients with severe and those with moderate or mild neurological sequelae; however, there was no significant difference between mild- and moderate-outcome groups [17]. Zhu et al. further described a significant association between severe neurological sequelae and more than two lesion locations as well as basal ganglia involvement [17]. Although more than two sites and part of the basal ganglia were involved in our reported case, the patient made a full recovery.

The sequences demonstrating pathological findings in our case were T2, FLAIR, diffusion weighted imaging (DWI), and SWI, which can be considered standard sequences in most MRI brain imaging protocols today. Interestingly, post-Gadolinium T1 sequences were normal in our patient, which is not uncommon in this disease, and this was also the case in 34% of patients with ANE in the study by Wong et al. [3]. In a case report of ANE in a 2-year-old boy by Yoshida et al. on the other hand, all non-contrast sequences were normal, and abnormal bilateral enhancement of the brainstem, thalami, and periventricular white matter was the only MRI finding, thus supporting post-Gadolinium imaging as an indispensable component of brain MRI protocols in such clinical settings, in particular at an early disease stage [18,19]. A recent study by Sarigecili et al. emphasised DWI as the crucial sequence in patients with suspected ANE, recommending that scans be routinely started with DWI in this clinical setting, especially given the risk of early abandonment of the examination in age-critical or severely ill patients [20].

Treatment for ANE mainly combines immunosuppression and supportive care, and to the best of our knowledge, there is currently no literature suggesting a differing treatment for patients with ANE1. A recently published systematic meta-analysis of outcomes with different immunosuppressive therapies in ANE described superior outcomes with early high-dose intravenous steroids, and no benefit of intravenous immunoglobulins was found [21]. There was a trend to suggest that tocilizumab may improve morbidity outcomes, especially when complementing early steroids [21]. Furthermore, plasma exchange therapy may be associated with increased survival [22]. Our patient received empirical dexamethasone within 12 h of presentation, which was escalated to intravenous methylprednisolone after the result of the MRI. Our patient’s ANE-SS was three (moderate), putting her at a medium risk of neurological sequelae, and early treatment with steroids likely significantly contributed to her good outcome and full recovery [14].

To date, positive *RANBP2* mutation status was found to be predictive of relapse but not predictive of the overall outcome [13]. Interestingly, there are multiple reported cases of milder neurological symptoms accompanying viral illnesses and not fulfilling the diagnosis of ANE in patients with a known *RANBP2* mutation, which potentially indicates milder courses of disease in this population on average [16,23]. The risk of disease recurrence in ANE1 is high, and up to 50% of patients experience at least two episodes [7]. Furthermore, this entity is likely underdiagnosed, and in the given context, our patient’s familial history of encephalitis in the maternal grandmother with full recovery is suspicious for an episode of ANE1 that did not receive any dedicated investigations. It is similarly probable that the maternal cousin’s death from encephalitis was a severe fatal episode of ANE1; albeit we could not obtain any further evidence in this regard. The patient’s family was referred for genetic counselling, which is currently still pending.

Study populations in research on ANE and even more so on ANE1 are generally small, and there are conflicting results regarding clinical and imaging factors and their possibly predictive value for patient outcomes in the literature to date [3,13,17,18,21]. Further clarifying research in this regard is thus warranted, potentially in multi-centre studies. Differentiating cases of ANE1 from ANE can improve management of the patient and their relatives. Awareness of the risk of recurrence could result in measures to avoid possible disease triggers, for example, through regular immunisations, and we encourage timely presentations to medical professionals when feeling significantly unwell.

## 4. Conclusions

We present a case of ANE1 with characteristic MRI findings. Recognition of the MRI involvement pattern in ANE1 can enhance management not only of the affected patient but also their relatives, thereby potentially improving clinical outcomes. Fortunately, our patient has fully recovered from her first episode of ANE1 without neurological sequelae, but she remains at risk for disease recurrence. The family has been referred for genetic counselling, and regular immunisations including influenza and COVID as well as a low threshold for hospital presentations in case of future illness were advised.

## Data Availability

The original contributions presented in this study are included in the article. Further inquiries can be directed to the corresponding author.
